# Development of Methanol Permselective FAU-Type Zeolite Membranes and Their Permeation and Separation Performances

**DOI:** 10.3390/membranes11080627

**Published:** 2021-08-15

**Authors:** Ayumi Ikeda, Chie Abe, Wakako Matsuura, Yasuhisa Hasegawa

**Affiliations:** Research Institute of Chemical Process Technology, National Institute of Advanced Industrial Science and Technology (AIST), 4-2-1 Nigatake, Miyagino-ku, Sendai 983-8551, Japan; a-ikeda@aist.go.jp (A.I.); abe-chie@aist.go.jp (C.A.); wakako.matsuura@aist.go.jp (W.M.)

**Keywords:** organic mixture separation, methanol removal, pervaporation

## Abstract

The separation of non-aqueous mixtures is important for chemical production, and zeolite membranes have great potential for energy-efficient separation. In this study, the influence of the framework structure and composition of zeolites on the permeation and separation performance of methanol through zeolite membranes were investigated to develop a methanol permselective zeolite membrane. As a result, the FAU-type zeolite membrane prepared using a solution with a composition of 10 SiO_2_:1 Al_2_O_3_:17 Na_2_O:1000 H_2_O showed the highest permeation flux of 86,600 μmol m^−2^ s^−1^ and a separation factor of 6020 for a 10 wt% methanol/methyl hexanoate mixture at 353 K. The membrane showed a molecular sieving effect, reducing the single permeation flux of alcohol with molecular size for single-component alcohols. Moreover, the permeation flux of methanol and the separation factor increased with an increase in the carbon number of the alcohols and methyl esters containing 10 wt% methanol. In this study, the permeation behavior of FAU-type zeolite membranes was also discussed based on permeation data. These results suggest that the FAU-type zeolite membrane has the potential to separate organic solvent mixtures, such as solvent recycling and membrane reactors.

## 1. Introduction

The separation of organic mixtures is essential for the chemical and petrochemical industries to improve process efficiency. In particular, organic solvent recycling, isomer separation, and the integration of reaction and separation processes have been reported [1]. Membrane separation is an energy-efficient separation technology, and inorganic membranes have superior chemical stability and mechanical strength compared to polymeric membranes. Among inorganic membranes, porous membranes, such as zeolite, silica, and carbon membranes, can separate organic mixtures by molecular sieving and selective adsorption.

Recently, the separation of organic mixtures using membranes by the molecular sieving effect has been reported [2,3,4]. Tsuru et al. developed a methanol permselective organosilica membrane using bis(triethoxysilyl)acetylene as a precursor [2]. The methanol flux was 24,300 μmol m^−2^ s^−1^ with a separation factor of 10 for a dimethyl carbonate mixture containing 10 wt% methanol at 323 K. Lively et al. examined xylene isomer separation by reverse osmosis method using carbon molecular sieve hollow fiber membranes derived from cross-linked poly(vinylidene fluoride) [3]. The flux was 1100 μmol m^−2^ s^−1^ with a separation factor of 4 for a xylene mixture (*o*-/*p*-xylene = 50/50) at 395 K. When an MFI-type zeolite membrane was used for xylene separation, the *p*-xylene flux was 2.3 μmol m^−2^ s^−1^ with a separation factor of more than 100 at 373 K [4]. The high permeation and separation performances of the zeolite membrane were achieved because the membrane had few defects such as pinholes and cracks.

Zeolite membranes can separate organic mixtures by selective adsorption, for example, alcohol/alcohol, alcohol/ether, and aromatic/alkane mixtures [5,6]. Kanemoto et al. evaluated the permeation performance of an MFI-type zeolite membrane for a mixture of 1-butanol, 2-propanol, and butyrate [5]. As a result, 1-butanol can selectively permeate because it has a higher carbon number and more hydrophobicity than 2-propanol. Kita et al. reported that an FAU-type zeolite membrane could separate benzene from cyclohexane [6]. The benzene selectivity was attributed to the selective adsorption of benzene on the zeolite by the interaction of the covalent electrons of benzene with Na^+^ in the zeolite. The FAU-type zeolite membrane was also effective for the separation of methanol from methyl tert-butyl ether.

Membrane reactors have attracted much attention for the development of energy- and resource-saving chemical reactions. The application of zeolite membranes to transesterification reactions has recently been investigated [7,8]. The selective removal of by-products (such as alcohol) from reaction mixtures can shift the chemical equilibrium, which improves the yield of the target ester. We investigated the influence of the reaction substrates of transesterification reactions on the conversion increment in the membrane reactor [9]. As a result, the permeation of methanol through the zeolite membrane significantly improved the yield of the target ester. However, it is unclear which zeolite frameworks and compositions are suitable for alcohol separation from non-aqueous organic solutions.

In this study, four zeolite membranes were prepared, and their permeation and separation performances of methanol from alcohols and methyl esters were evaluated to determine the influence of the framework structure and composition of the zeolite membrane. Since the FAU-type zeolite showed the highest permeation and separation performance of methanol among the membranes, the permeation performances were investigated for single-component alcohols and several organic solvents containing methanol. Furthermore, the permeation and separation behavior of the FAU-type zeolite membrane are discussed based on these data in this manuscript.

## 2. Materials and Methods

### 2.1. Materials

Sodium aluminate, sodium hydroxide, sodium silicate solution, tetrapropylammonium bromide (TPABr), N,N,N-trimethyl-1-adamantammonium hydroxide (TMAdOH) solution, colloidal silica (LUDOX HS-40), NaY-type zeolite particles (HSZ-320NAA, Si/Al = 2.3) and HY-type zeolite particles (HSZ-390HUA, Si/Al = 770) were used for the preparation of zeolite membranes. Methanol, ethanol, 1-propanol, 1-butanol, 1-hexanol, methyl acetate, methyl propionate, methyl butyrate, and methyl hexanoate were used in the pervaporation experiments. All reagents were purchased from FUJIFILM Wako (Osaka, Japan) and used without further purification.

### 2.2. Membrane Preparation

CHA-, LTA-, MFI-, and FAU-type zeolites were selected as the potential material for methanol permselective membranes. CHA- and LTA-type zeolites have similar pore diameters (0.38, 0.42 nm) to the molecular size of methanol (0.380 nm). Besides, MFI- and FAU-type zeolite membranes can separate alcohol from alcohol/ester mixtures [5] and methanol/organic solvent mixture separation [6], respectively. Therefore, we selected the above four types of zeolite membranes.

Four types of zeolite membranes (CHA-, LTA-, MFI-, and FAU-type) were synthesized on the outside of porous α-alumina support tubes by a secondary growth method. The properties of the support tube were as follows: Diameter = 3 mm, mean pore diameter = 0.3 μm, and porosity = 50%. The membranes were prepared using the same method as previously reported [10,11,12]. The detailed preparation procedures for zeolite membranes are described as follows.

For the CHA-type zeolite membrane [10], a synthesis solution was prepared by mixing sodium hydroxide, sodium aluminate, TMAdOH solution, and HY-type zeolite particles, followed by stirring for 4 h at room temperature. The molar composition of the mixture was 45 SiO_2_:1 Al_2_O_3_:4.5 Na_2_O:3.2 TMAdOH:4500 H_2_O. The CHA-type zeolite particles were manually implanted into the macropores of the support tube by rubbing with paper wipers. The tube was added to a Teflon-lined stainless-steel autoclave filled with 30 g of the synthesis solution. The autoclave was placed horizontally in an oven at 433 K for 24 h to form a polycrystalline layer on the support. After the autoclave was cooled to room temperature, the tube was removed from the autoclave and washed with deionized water several times. The tube was dried overnight at room temperature and then fired at 773 K for 10 h to obtain the CHA-type zeolite membrane (hereafter referred to as the CHA membrane).

For the LTA-type zeolite membrane [11], a synthesis solution was prepared by stirring a mixture of sodium aluminate, sodium hydroxide, and sodium silicate solution for 1 h at room temperature. The molar composition of the mixture was 2 SiO_2_:1 Al_2_O_3_:2.3 Na_2_O:300 H_2_O. The outside of the support tube was rubbed with LTA-type zeolite particles, and the tube was added to an autoclave filled with 30 g of the synthesis solution. The autoclave was placed horizontally in an oven at 393 K for 5 h. After the reaction, the autoclave was cooled to room temperature, and the tube was recovered and washed with deionized water several times. Finally, the membrane was dried overnight under ambient conditions to obtain the LTA-type zeolite membrane (hereafter referred to as the LTA membrane).

For the MFI-type zeolite membrane [12], a synthesis solution was prepared by mixing sodium hydroxide, colloidal silica, deionized water, and TPABr as a structure-directing agent, followed by stirring for 1 h at room temperature. The molar ratio of the synthesis solution was 1 SiO_2_:0.05 Na_2_O:0.26 TPABr:80 H_2_O. The outside of the support tube was rubbed with MFI-type zeolite particles, and both ends of the tube were sealed with Teflon tape to prevent crystal deposition on the inner surface of the tube. The tube was then placed in a Teflon-lined stainless-steel autoclave filled with 30 g of the synthesis solution. The autoclave was placed horizontally in an oven at 413 K for 24 h. After the autoclave was cooled to room temperature, the tube was removed from the autoclave and washed with deionized water several times. The tube was dried overnight at room temperature and then fired at 673 K for 48 h to obtain the MFI-type zeolite membrane (hereafter referred to as the MFI membrane).

For the FAU-type zeolite membrane [12], a synthesis solution was prepared by stirring a mixture of sodium hydroxide, sodium aluminate, sodium silicate solution, and deionized water for 4 h at room temperature. The molar composition of the mixture was *a* SiO_2_:1 Al_2_O_3_:17 Na_2_O:1000 H_2_O (*a* = 5, 10, 13, and 25). The NaY-type zeolite particles were rubbed on the outside of the support tube, and the tube was added to an autoclave filled with 30 g of the synthesis solution. The autoclave was set horizontally in an oven at 363 K for 16 h to grow the FAU-type zeolite crystals on the support tube. The tube was then washed with deionized water several times after cooling the autoclave and dried overnight at room temperature. Hereafter, the FAU-type zeolite membranes with molar compositions of *a* = 5, 10, 13, and 25 are referred to as FAU(5), FAU(10), FAU(13), and FAU(25) membranes, respectively.

The membrane morphology was observed using scanning electron microscopy (SEM, JEOL, JCM-6000, Tokyo, Japan), and the composition was analyzed using an energy dispersive X-ray analyzer (EDX, JEOL, ED-2300, Tokyo, Japan) attached with the SEM. The crystal structure was identified by X-ray diffraction (XRD, Rigaku, Smart-Lab, Tokyo, Japan).

### 2.3. Pervaporation Experiment

**Figure 1:**
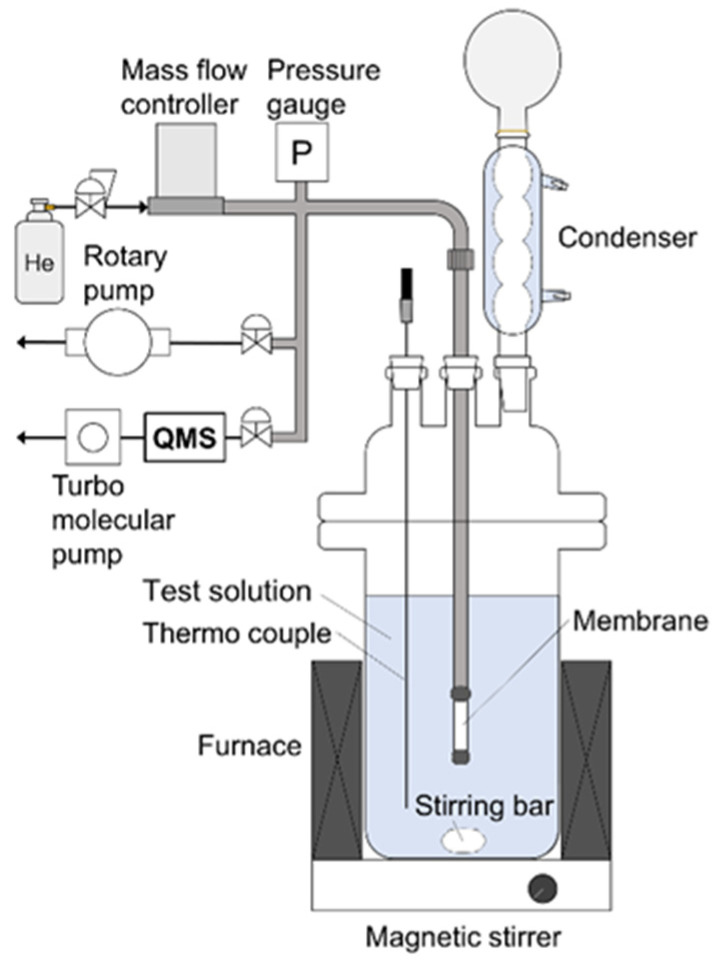
Schematic diagram of pervaporation apparatus.

One end of the membrane was connected to a stainless-steel tube using a resin (GL Science, Torr seal, Tokyo, Japan), and the other end was capped. The effective membrane areas were 1.0 cm^2^ for the CHA, LTA, and MFI membranes and 2.0 cm^2^ for the FAU membrane, respectively. All membrane performances were evaluated by a pervaporation method using the apparatus shown in Figure 1. Ethanol, 1-propanol, 1-butanol, 1-hexanol, methyl acetate, methyl propionate, methyl butyrate, and methyl hexanoate were used as solvents in the test solution containing 10 wt% methanol for the pervaporation experiment. These alcohols and methyl esters are used as reaction substrates of transesterification reaction in our previous report [9]. The test solution (150 g) was added to a separable flask and heated at 303–353 K with stirring at 600 rpm. The membrane was immersed in the solution, and the inside of the membrane was evacuated using a rotary pump below 1 kPa. In addition, helium was introduced into the membrane at 1.0, 3.0, or 6.0 mL min^−1^ as the standard. The gas composition in the evacuated stream was analyzed using mass spectrometry (Pfeiffer vacuum, QGA, Asslar, Germany). The permeation flux, *J_i_*, of component *i* was calculated as follows [13]:

(1) $$J_{i}=\frac{N_{\mathrm{He}}}{S}\frac{y_{i}}{y_{He}},$$

where *N*_He_, *S*, and *y*_i_ are the molar flow rate of helium, the membrane area, and the mole fraction of component *i* in the evacuated stream, respectively. The permeance of single alcohol, *Q_i_*, component, was calculated using the following equation:

(2) $$Q_{i}=\frac{J_{i}}{p_{f,i}-p_{p,i}},$$

where *p_f,i_* and *p_p,i_* represent the saturated vapor pressure of component *i* of the feed solution and the partial vapor pressure of component *i* on the permeate side, respectively. The saturated vapor pressure of component *i* was calculated using the Antoine constants listed in Table 1. The separation factor of methanol for each organic solvent, *α*, is defined as follows:

(3) $$\alpha_{\left(M/O\right)}=\frac{y_{M}/y_{O}}{x_{M}/x_{O}},$$

where *x_i_* is the mole fraction of component *i* in the solution, and the subscripts M and O indicate methanol and organic solvent, respectively.

### 2.4. Seed Particles Preparation for Zeolite Membrane

For the CHA membrane, the seed particles were prepared by mixing sodium hydroxide, sodium aluminate, N,N,N-trimethyl-1-adamantammonium hydroxide solution (SDA, 25%, Sachem Asia, Osaka, Japan), and ultra-stable Y-type zeolite particles (HSZ-390HUA). The molar composition of the solution was 40 SiO_2_:1Al_2_O_3_:4 Na_2_O:8 SDA:800 H_2_O. The mixture was poured into a Teflon-lined stainless-steel autoclave, and a hydrothermal reaction was carried out at 433 K for four days. Solids were recovered by filtration, washed with distilled water, and dried overnight at 383 K to obtain seed particles.

For the MFI membrane, the seed particles were prepared by mixing sodium hydroxide, tetrapropylammonium hydroxide (TPAOH), and tetraethyl orthosilicate. The molar composition of the solution was 25 SiO_2_:0.1 Na_2_O:9 TPAOH:490 H_2_O:100 EtOH. The mixture was poured into a Teflon-lined stainless-steel autoclave, and a hydrothermal reaction was carried out at 373 K for 72 h. Solids were recovered centrifugally and washed with distilled water. The average particle size was 110 nm.

Commercially available NaA (A-4) and NaY-type zeolite particles (HSZ-320NAA) were used as the seed particles in this study. They can be purchased from FUJIFILM Wako Corp (Osaka, Japan).

## 3. Results and Discussions

### 3.1. Membrane Characterization

**Figure 2:**
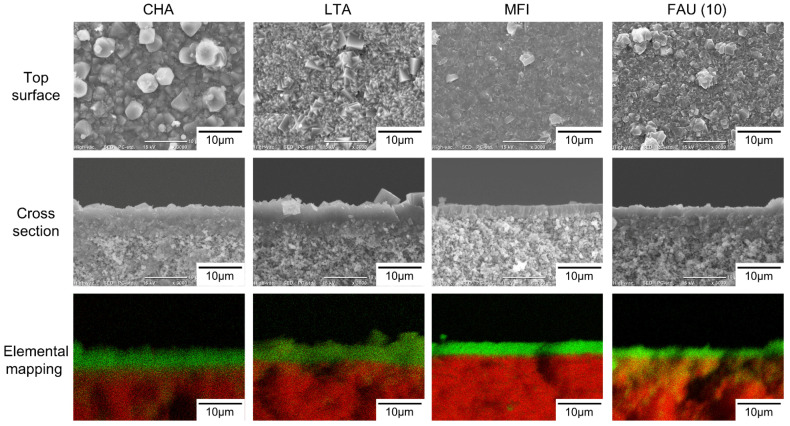
SEM images and cross-section elemental mapping of CHA, LTA, MFI, and FAU(10) membranes (Green: Si, Red: Al).

**Figure 3:**
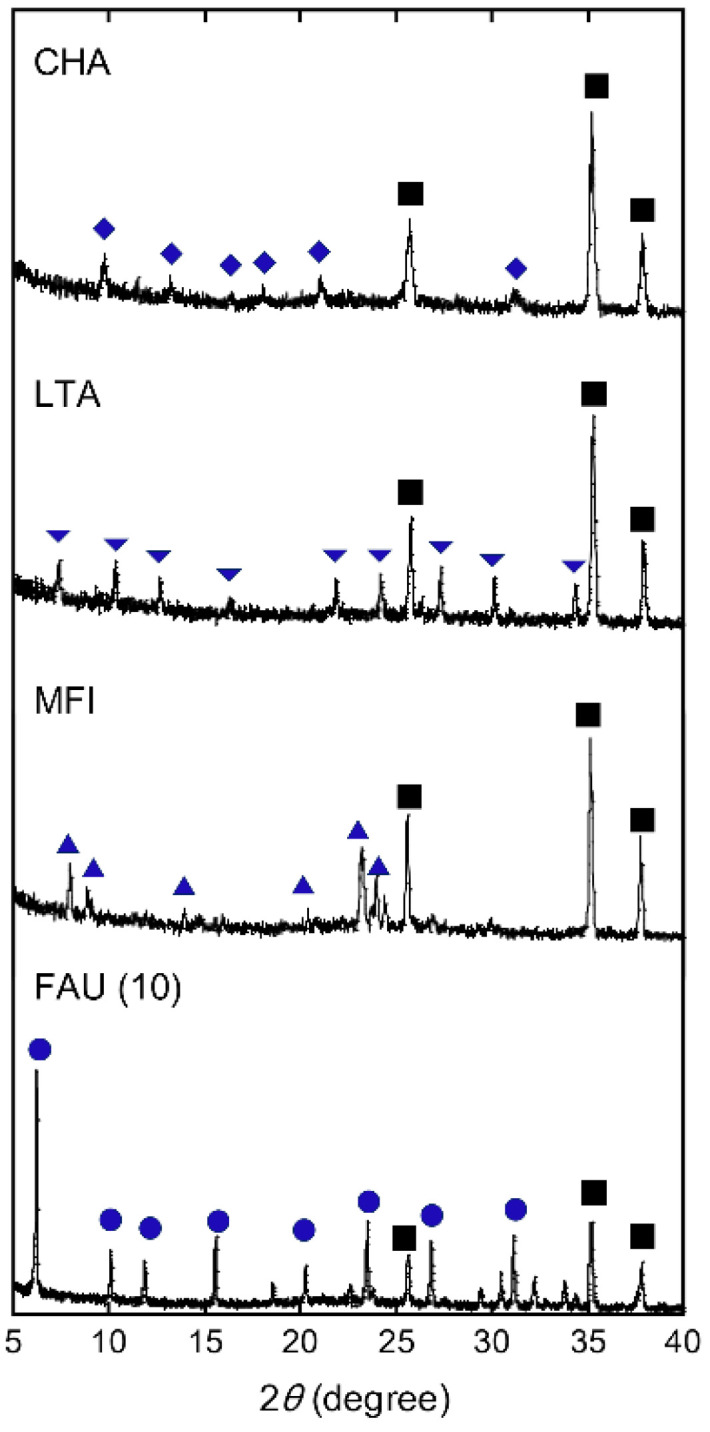
XRD patterns of CHA, LTA, MFI, and FAU(10) membranes. Symbols denote the diffraction peaks of CHA (diamond), LTA (inverted triangle), MFI (triangle), FAU (circle), and α-alumina (square).

Figure 2 shows the SEM images of the top surface and cross-section and elemental mapping by EDX of the CHA, LTA, MFI, and FAU(10) membranes. The outer surface of the support was covered with well-intergrown polycrystalline layers with sizes of 2–5 μm for CHA, 3–4 μm for LTA, 2–3 μm for MFI, and 1–4 μm for FAU. The thicknesses of the polycrystalline layers were in the range of 1.2–4.5 μm. The Si/Al ratios of CHA, LTA, MFI, and FAU(10) membranes were 18, 1.0, 75, and 1.3. by EDX. The values of CHA, LTA, and FAU(10) were similar to theoretical Si/Al ratios, while that of the MFI membrane was different from the theoretical Si/Al ratio (=∞). This proposes the zeolite layer contains aluminum derived from the α-alumina support. As shown in Figure 3, the XRD patterns of the membranes contain both the α-alumina support and desired zeolite. Table 2 shows major XRD peaks of four zeolites and the relative intensities. For CHA, the peaks due to (1,0,0), (1,0,−1), and (2,−1,0) were observed at 2*θ* = 9.5, 12.9, and 20.6°, respectively. LTA, MFI, and FAU membranes also give peaks due to corresponding zeolites. These results indicate that CHA, LTA, MFI, and FAU(10) membranes can be prepared on the supports. The peak intensity ratios due to zeolites were almost the same as those of the particles. This means that the zeolite crystals forming the polycrystalline layer are not oriented in a unique direction.

**Figure 4:**
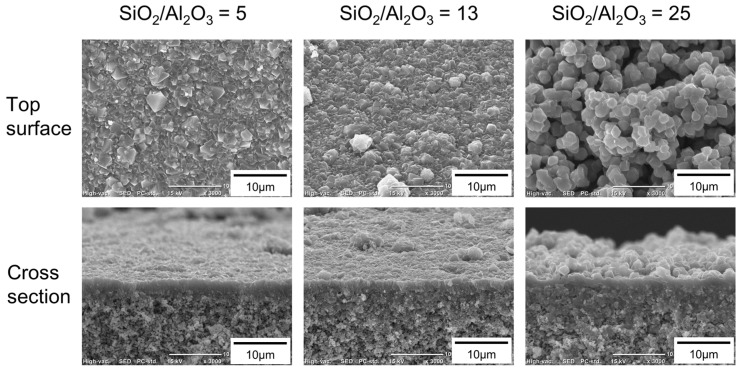
SEM images of FAU membranes prepared using the synthesis solutions with SiO_2_/Al_2_O_3_ ratios of 5, 13, and 25.

Figure 4 shows the effect of the molar composition of the synthesis solution on the morphology of the FAU membranes. When the molar composition of the synthesis solution was 25 SiO_2_:1 Al_2_O_3_:17 Na_2_O:1000 H_2_O (SiO_2_/Al_2_O_3_ = 25), many particles were deposited on the outer surface. However, few particles were observed at SiO_2_/Al_2_O_3_ ratios of 5, 10, and 13. The thicknesses of the polycrystalline layers were almost the same as 1.7–2.5 μm for all SiO_2_/Al_2_O_3_.

**Figure 5:**
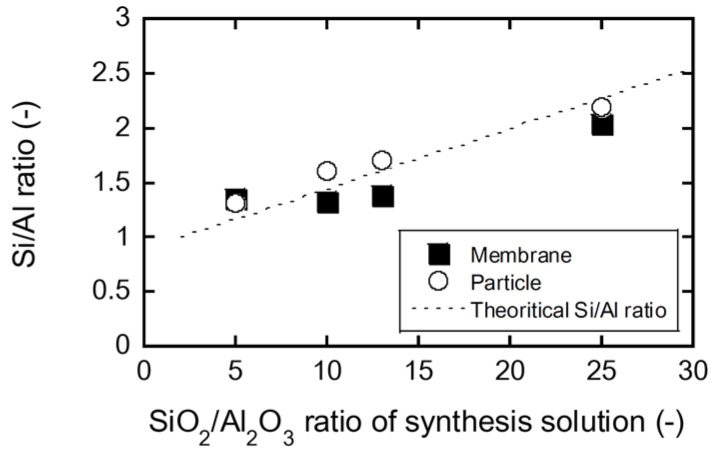
Influence of SiO_2_/Al_2_O_3_ ratio of synthesis solution on the Si/Al ratio of FAU membranes and particles.

Figure 5 shows the influence of the SiO_2_/Al_2_O_3_ ratio of the synthetic solution on the Si/Al ratios of the FAU zeolite membrane and the recovered particles. At SiO_2_/Al_2_O_3_ = 5, the Si/Al ratios of the particles and membrane were 1.35 and 1.31, respectively. As the SiO_2_/Al_2_O_3_ ratio of the solution increased, the Si/Al ratios of the particles and membrane also increased.

Lechert et al. investigated the influence of the molar composition of the synthesis solutions on the Si/Al ratio of FAU-type zeolite crystallites [16]. Assuming that the molar composition of the zeolite synthesis solution is *a* SiO_2_:1 Al_2_O_3_:*c* Na_2_O:*d* H_2_O, the theoretical Si/Al ratio of FAU-type zeolite crystallites can be described as follows:

(4) $$\mathrm{Si}/\mathrm{Al}=\frac{a+c-1}{c+1}.$$

The calculation result is represented by the dashed line in Figure 5. The good agreement between the experimental data and theoretical values indicates that the membrane composition can be controlled by the composition of the synthesis solution.

### 3.2. Methanol Separation Performance of CHA, LTA, MFI, and FAU Membranes

Table 3 shows the results of pervaporation experiments for a 10 wt% methanol/methyl hexanoate mixture at 353 K. For all the zeolite membranes, methanol permeated selectively, and the permeation fluxes of methyl hexanoate were less than 32 µmol m^−2^ s^−1^. The lower fluxes indicate that there are few large pinholes in the zeolite membranes.

The FAU(10) membrane with a large pore size showed an excellent methanol permeation flux of 86,600 µmol m^−2^ s^−1^ (=9.9 kg m^−2^ h^−1^) and a high separation factor of 6020. The reason was considered that FAU-type zeolite has the largest pore size (0.74 nm) among the tested zeolite membranes. The methanol selectively permeates through the FAU(10) membrane by selective adsorption and molecular sieving as discussed later (Figures 7 and 9). Because the pore diameter of the CHA-type zeolite (0.38 nm) is close to the molecular size of methanol (0.380 nm), the separation factor of the CHA membrane was as high as 1270. However, the permeation fluxes of the LTA and MFI membranes were lower than 1300 µmol m^−2^ s^−1^. The lower permeation fluxes could be attributed to the lower adsorption capacity of the LTA-type zeolite [17] and the inhibition of methanol diffusion by methyl hexanoate for MFI-type zeolite [18].

### 3.3. Effect of Si/Al Ratio of FAU Membrane

Next, the effect of the composition of the synthesis solution on the separation and permeation performance of methanol through the FAU membrane was investigated. Table 4 shows the effect of the SiO_2_/Al_2_O_3_ ratio on the permeation performance of the FAU membranes for the 10 wt% methanol/methyl hexanoate mixture at 353 K. When SiO_2_/Al_2_O_3_ = 5, the methanol permeation flux and separation factor were 51,900 µmol m^−2^ s^−1^ and 3890, respectively. As the SiO_2_/Al_2_O_3_ ratio of the synthetic solution increased, the permeation flux increased, reaching 184,000 μmol m^−2^ s^−1^ at SiO_2_/Al_2_O_3_ = 25. In contrast, a maximum separation factor of 6020 was obtained for SiO_2_/Al_2_O_3_ = 10. The FAU(13) and (25) membranes were supposed to exist pinholes since the permeation fluxes of methyl hexanoate were two orders of magnitude higher. Especially, the FAU(25) membrane observed a lot of particles on the surface (Figure 4), so it indicated the formation of zeolite particles was superior to the membrane formation at SiO_2_/Al_2_O_3_ = 25, whereas the FAU(5) and (10) membranes had few pinholes, since the permeation fluxes of methyl hexanoate were low. By increasing the SiO_2_/Al_2_O_3_ ratio from 5 to 10, the hydrophobicity of the FAU(10) membrane could be increased. As the result, the permeation flux of methanol through the FAU(10) membrane was obtained 1.7 times higher than that of the FAU(5) membrane.

### 3.4. Single Alcohol Permeation Performance

**Figure 6:**
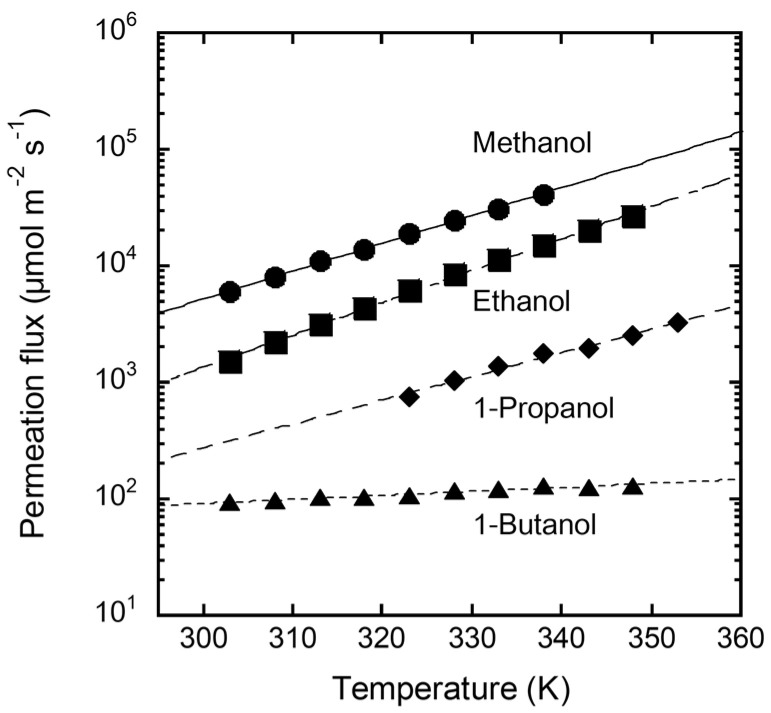
Temperature dependency of permeation fluxes of single alcohols through the FAU(10) membrane.

Figure 6 shows the temperature dependence of the permeation fluxes of single-component alcohols through the FAU(10) membrane. The methanol permeation flux was 5900 µmol m^−2^ s^−1^ at 303 K. The permeation flux decreased as the carbon number increased, and that of 1-butanol was 94.4 µmol m^−2^ s^−1^ at 303 K. The permeation flux of methanol increased with increasing solution temperature, reaching 41,400 µmol m^−2^ s^−1^ at 338 K. Ethanol and 1-propanol showed temperature dependencies similar to that of methanol. In contrast, the effect of temperature on the permeation flux of 1-butanol was smaller than that of the other alcohols.

**Figure 7:**
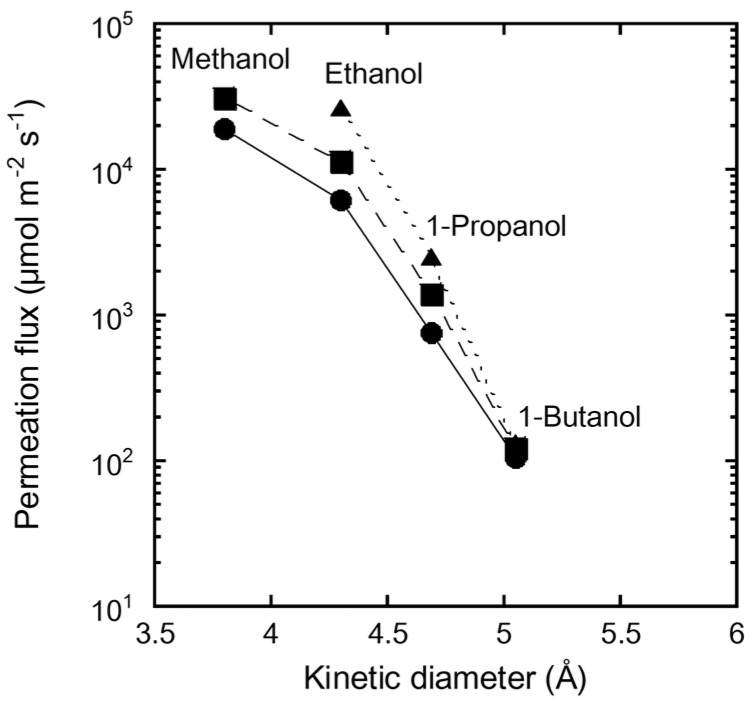
Influence of kinetic diameter of alcohol on permeation flux of single alcohol through the FAU(10) membrane at 323 K (circle), 333 K (square), and 348 K (triangle).

Figure 7 shows the influence of the molecular diameter on the permeation flux of single-component alcohols through the FAU(10) membrane at 323, 333, and 348 K. The permeation flux of methanol was 31,100 µmol m^−2^ s^−1^ at 333 K. The flux decreased with increasing molecular diameter, and that of 1-butanol was 122 µmol m^−2^ s^−1^. This significant effect of molecular size on the permeation flux is attributed to the molecular sieving function of the zeolite, even though the pore size of the FAU-type zeolite (0.74 nm) is larger than the molecular diameters of the alcohols (ethanol: 0.430 nm, 1-propanol: 0.469 nm, and 1-butanol: 0.504 nm). These results also support our claim that there are no large pinholes in the FAU membranes, as discussed in Table 3.

Because the permeation flux is described as the product of the permeance and the partial pressure difference, the permeation flux in Figure 6 includes the temperature dependence of the vapor pressure. To discuss only the membrane permeation phenomenon, the permeation flux was converted to permeance. The temperature dependence of the vapor pressure was calculated using the Antoine constant (Table 1).

**Figure 8:**
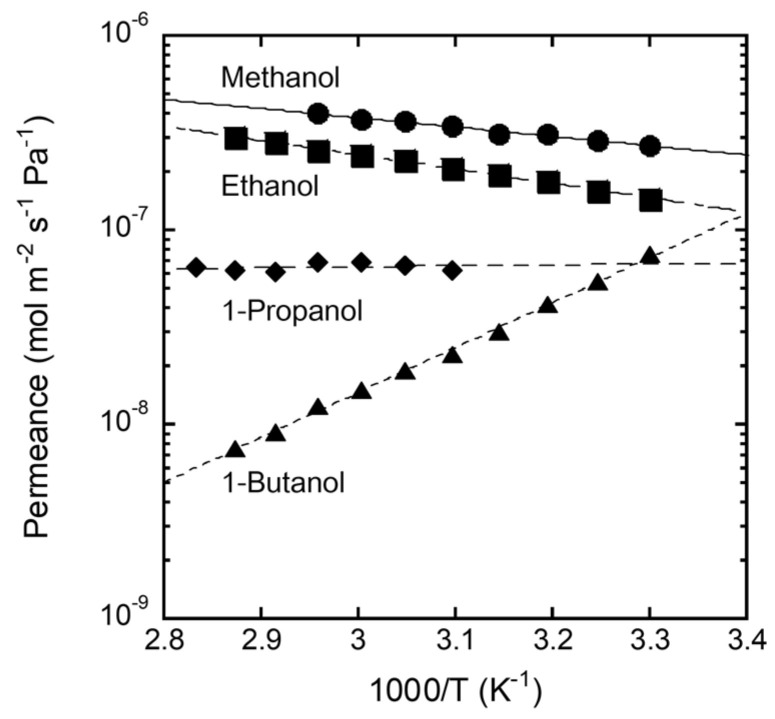
Arrhenius plot of permeances of single alcohols through the FAU(10) membrane (the activation energy for permeation: Methanol; 9.1 kJ mol^−1^, ethanol; 14.1 kJ mol^−1^, 1-propanol; −0.9 kJ mol^−1^, 1-butanol; −44.2 kJ mol^−1^).

Figure 8 shows the Arrhenius plot of the single-component alcohol permeance through the FAU(10) membrane. The permeance of methanol was 2.7 × 10^−^^7^ mol m^−2^ s^−1^ Pa^−1^ at 303 K, increased with temperature, and reached 4.0 × 10^−7^ mol m^−2^ s^−1^ Pa^−1^ at 338 K. The activation energy for permeation was 9.1 kJ mol^−1^ by calculation with the Arrhenius equation. The permeance of ethanol also showed a similar temperature dependence to methanol, with an activation energy for permeation of 14.1 kJ mol^−1^. However, 1-Butanol showed the reverse trend, with an activation energy for permeation of −44.2 kJ mol^−1^. The effect of temperature on the permeance of 1-propanol was small (activation energy for permeation = −0.9 kJ mol^−1^).

The molecules adsorbed in the pores diffuse across the zeolite membrane according to the concentration gradient. The adsorbed amount decreased as the temperature increased, while the diffusion coefficient increased inversely. The activation energy for permeation is expressed as the sum of the heat of adsorption and the activation energy for diffusion. This suggests that the permeation of methanol and ethanol with positive activation energies was strongly influenced by diffusion, while adsorption had a significant effect on the permeation of 1-butanol. It is understood that 1-butanol is difficult to adsorb into the zeolite pores, as considering the results in Figure 6.

### 3.5. Permeation and Separation Performance of Methanol from Methanol/Organic Solvent Mixture

Table 5 shows the influence of organic solvent species on the permeation and separation performance of methanol through the FAU(10) membrane. When methanol was mixed with ethanol, the methanol permeation flux was 14,700 μmol m^−2^ s^−1^ and the separation factor was 8 at 348 K. These increased with the carbon number of alcohols mixed with methanol; thus, for 1-hexanol, the methanol permeation flux and separation factor reached 51,200 µmol m^−2^ s^−1^ and 980, respectively. The same trend was observed when methanol was mixed with methyl esters (except in the case of methyl butyrate).

**Figure 9:**
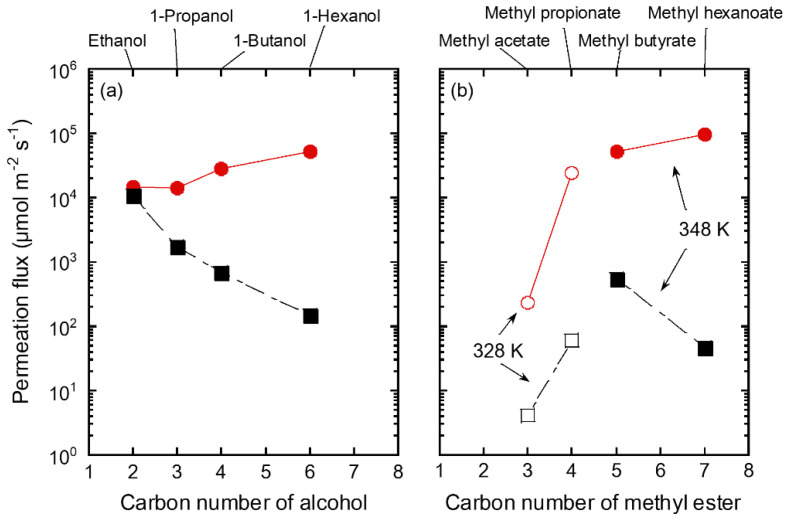
Influence of carbon number of (**a**) alcohol and (**b**) methyl ester on permeation fluxes of the FAU(10) membranes for 10 wt% methanol/solvent mixtures at 328 K (unfilled) and 348 K (filled). Symbols denote methanol (circle) and solvents (square).

Figure 9 shows the influence of the carbon number of the organic solvent on the permeation fluxes of methanol and solvents for the FAU(10) membrane. As mentioned in Table 5, the permeation flux of methanol increased with the carbon number of alcohols, while that of mixed alcohols showed the reverse tendency. Even when methyl esters were mixed with methanol, the permeation flux of methanol showed a similar increasing trend, although the measurement temperature was different.

As discussed in Figure 8, methanol and ethanol molecules adsorbed on the zeolite diffuse into the pores and permeate through the zeolite membrane according to the concentration gradient. Therefore, for a mixed solution of methanol and ethanol, the permeation of methanol was inhibited by ethanol in the zeolite pores, which reduced the permeation flux of methanol. In contrast, the adsorption of 1-butanol into zeolite pores is important for the permeation of 1-butanol, as shown in Figure 8. For a mixture of methanol and 1-butanol, the permeation of methanol is not inhibited by 1-butanol because it is difficult for 1-butanol to adsorb on the zeolite pores. As a result, both the permeation flux and separation factor improved for 1-hexanol and methyl hexanoate with more carbons than 1-butanol.

## 4. Conclusions

For developing a methanol permselective zeolite membrane, we investigated the influence of the framework structure, composition, and organic compounds on the permeation and separation performance of methanol through zeolite membranes in this study. First, CHA, LTA, MFI, and FAU membranes with few pinholes were successfully prepared on porous support tubes. Then, the permeation and separation performances of methanol were determined by pervaporation with a 10 wt% methanol/methyl hexanoate mixture. As a result, the FAU membrane synthesized with a composition of 10 SiO_2_:1Al_2_O_3_:17 Na_2_O:1000 H_2_O showed a high permeation flux of 86,600 μmol m^−2^ s^−1^ and a separation factor of 6020 at 353 K. Next, FAU membranes were prepared using synthetic solutions with different SiO_2_/Al_2_O_3_ ratios to investigate the influence of the zeolite composition on the permeation and separation performances of methanol through the FAU membranes. The highest separation factor was obtained at SiO_2_/Al_2_O_3_ = 10, although the permeation flux of methanol increased with a higher SiO_2_/Al_2_O_3_ ratio. The FAU membrane showed a molecular sieving effect that reduced the single permeation flux of alcohol with molecular size. Moreover, diffusion affected the permeation of methanol and ethanol through the FAU membrane, while adsorption significantly affected the permeation of 1-butanol. Finally, pervaporation experiments using the FAU membrane were carried out for several alcohols and methyl esters. As a result, the permeation flux of methanol increased with the carbon number of organic solvents because of the reduction in the inhibition of methanol permeation by these organic solvents. These results suggest that the FAU membrane has the potential to separate organic mixtures, such as solvent recycling and membrane reactors.

## Figures and Tables

**Table 1:** Antoine constants of primary alcohols.

Solvent	Antoine Constant			Reference
	A	B	C	
Methanol	8.07919	1581.34	239.65	[14]
Ethanol	8.04494	1554.3	222.65	[14]
1-Propanol	7.751113	1441.6293	198.8507	[14]
1-Butanol	4.54607	1351.555	−93.34	[15]

**Table 2:** XRD peaks and relative intensity, *I*_rel_, for CHA, LTA, MFI, and FAU(10) particles.

CHA			LTA			MFI			FAU(10)		
2*θ* (°)	(*h*,*k*,*l*)	*I*_rel_ (%)	2*θ* (°)	(*h*,*k*,*l*)	*I*_rel_ (%)	2*θ* (°)	(*h*,*k*,*l*)	*I*_rel_ (%)	2*θ* (°)	(*h*,*k*,*l*)	*I*_rel_ (%)
9.5	(1,0,0)	100	7.2	(2,0,0)	100	7.9	(0,1,1)	100	6.2	(1,1,1)	100
12.9	(2,−1,0)	47.2	10.2	(2,2,0)	76.9	8.9	(2,0,0)	59.7	15.6	(3,3,1)	33.8
20.6	(1,0,−1)	50.4	16.1	(4,2,0)	36.2	23.1	(0,5,1)	110	23.6	(5,3,3)	30.7

**Table 3:** Permeation and separation performances of methanol through zeolite membranes for 10 wt% methanol/methyl hexanoate mixture at 353 K.

Zeolite	Flux (μmol m^−2^ s^−1^)		Separation Factor (-)
	Methanol	Methyl Hexanoate	
CHA	13,800	24.0	1270
LTA	1070	10.5	225
MFI	1300	17.2	168
FAU(10)	86,600	31.9	6020

**Table 4:** Effect of SiO_2_/Al_2_O_3_ ratio of synthesis solutions on the permeation performances of the FAU zeolite membranes for 10wt% methanol/methyl hexanoate mixture at 353 K.

SiO_2_/Al_2_O_3_ Ratio (-)	Flux (μmol m^−2^ s^−1^)		Separation Factor (-)
	Methanol	Methyl Hexanoate	
5	51,900	29.5	3890
10	86,600	31.9	6020
13	106,000	1520	155
25	184,000	2710	150

**Table 5:** Influence of solvent species on permeation and separation performances of methanol through the FAU(10) membrane for 10 wt% methanol/solvent mixtures.

Solvent	Temperature (K)	Flux (μmol m^−2^ s^−1^)		Separation Factor (-)
		Methanol	Solvent	
Ethanol	348	14,700	11,000	8
1-Propanol	348	14,400	1740	40
l-Butanol	348	27,900	695	160
1-Hexanol	348	51,200	148	980
Methyl acetate	328	231	4.2	510
Methyl propionate	328	24,400	62.0	1290
Methyl butyrate	348	52,300	538	270
Methyl hexanoate	348	98,200	47.0	4630
